# Pathogenic variants in the polycystin pore helix cause distinct forms of channel dysfunction

**DOI:** 10.1073/pnas.2421362122

**Published:** 2025-06-12

**Authors:** Orhi Esarte Palomero, Eduardo Guadarrama, Paul G. DeCaen

**Affiliations:** ^a^Department of Pharmacology, Feinberg School of Medicine, Northwestern University, Chicago, IL 60061; ^b^The Chemistry of Life Processes Institute, Northwestern University, Evanston, IL 60208

**Keywords:** autosomal polycystic kidney disease, cryo-EM, polycystin, TRP channels

## Abstract

PKD2 is a member of the polycystin subfamily of transient receptor potential (TRP) ion channel subunits which traffic and function in primary cilia organelle membranes. Millions of individuals carry pathogenic genetic variants in PKD2 that cause a life-threatening condition called autosomal dominant polycystic kidney disease (ADPKD). Although ADPKD is a common monogenetic disorder, there is no drug cure or available therapeutics which address the underlying channel dysregulation. Furthermore, the structural and mechanistic impacts of most disease-causing variants are uncharacterized. Using direct cilia electrophysiology, cryogenic electron microscopy (cryo-EM), and superresolution imaging, we have found mechanistic differences in channel dysregulation caused by three germline missense variants located in PKD2’s pore helix 1. Variant C632R reduces protein thermal stability, resulting in impaired channel assembly and abolishes primary cilia trafficking. In contrast, variants F629S and R638C retain native cilia trafficking but exhibit gating defects. Cryo-EM structures (2.7 to 2.8 Å resolution) indicate loss of critical pore helix interactions which precipitate allosteric collapse of the channels inner gate. Results demonstrate how ADPKD-causing mutations cause mechanistically divergent and ranging impacts on PKD2 function, despite their shared structural proximity. These unexpected findings highlight the need for structural and biophysical characterization of polycystin variants, which will guide rational drug development of ADPKD therapeutics.

## Significance

ADPKD is a common monogenetic disorder—impacting millions of individuals worldwide. The disease is initiated by variants in renal polycystins (PKD1 and PKD2), which function as ion channel subunits in the primary cilia. Yet, the impact of disease-causing variants on polycystin structure and function is poorly understood. Using cutting-edge approaches, we find mechanistic differences and divergent effects on channel function, assembly, and cilia trafficking caused by pore helix variants. Resolved cryo-EM structures demonstrate how some mutations cause long ranging collapse of the channel’s internal gate, while others impair assembly. The unexpected findings have significant implications for rational drug development of ADPKD therapeutics and our understanding of allosteric regulation of polycystin conductive states.

Renal polycystins (PKD1 and PKD2) are ion channel subunits with associated genetic variants that cause autosomal dominant polycystic kidney disease (ADPKD) (1–3). ADPKD is a lethal monogenetic disorder characterized by progressive cyst development that precipitates renal failure (4, 5). More than 12 million individuals carry disease-causing variants in PKD1 or PKD2, which accounts for the majority (>95%) of the clinically reported cases of ADPKD (4). Although ADPKD is commonly considered a loss-of-function disease putatively driven by haploinsufficiency or defective protein expression, the mechanistic impact of most polycystin variants remains uncharacterized (5, 6). There is no cure for ADPKD, and current treatments focus on managing symptoms and indirect means of slowing disease progression (7, 8). Thus, defining the structural and functional impact of disease-causing polycystin variants is critical to inform the development of targeted therapies.

ADPKD is considered a “channelopathy” and a “ciliopathy”—meeting both criteria for a disease that is caused by ion channel variants that precipitate ciliary dysregulation (9, 10). Renal polycystins traffic to the primary cilium of collecting duct principal cells, where they form homomeric (PKD2) and heteromeric channel complexes (PKD1-PKD2) (11–13). While the characteristics of native heteromeric PKD1-PKD2 channel conductance are poorly understood, recent work suggests that the complex is not constitutively active, rather is activated after cleavage and/or binding of PKD1’s N terminus (11). In contrast, homomeric PKD2 channels are voltage-gated, outwardly rectifying and Ca^2+^ modulated when assessed directly from the primary cilia membrane using submicron diameter electrodes (12–14). Primary cilia are small (d ~300 nm, l ~ 2 to 12 µm), singular projections that are highly insulated from the cell body (15). PKD2 contributions to plasma membrane current are nominal when assessed natively in kidney principal cells or when the channel is overexpressed (12). While the high-resolution cryogenic electron microscopy (cryo-EM) structures of homomeric polycystins have provided a molecular bases for the present study, our understanding of the structural motifs responsible for channel function and assembly is limited (16–19). Given their tenability to structural analysis using cryo-EM and functional analysis within the primary cilia, we will primarily consider the impact of PKD2 variants within the context of homomeric channels.

Previously, we used direct cilia electrophysiology, and cryo-EM structure determination to define how variants in the PKD2 TOP domain cause local structural destabilization and gating defects (20). In this manuscript, we apply these techniques along with superresolution cilia trafficking imaging analysis to evaluate pathogenic PKD2 (https://pkdb.mayo.edu/variants) germline missense variants (F629S, C632R, R638C) found within pore helix 1 (PH1) of the channels pore domain (*SI Appendix*, Fig. S1) (21). Our results define mechanistic differences among these loss-of-function variants, despite impacting the same structural motif within the PKD2 channel. Alterations in the variant cryo-EM structures and their biophysical gating defects implicate a long-range allosteric mechanism controlling the opening of the channel pore. Significance of the findings underscores the need to further characterize the diversity in polycystin variant molecular dysfunction to guide rational approaches for ADPKD drug development.

## Results

### Destabilizing Effects of Pore Helix Variants on Channel Assembly and Structure.

**Fig. 1.**
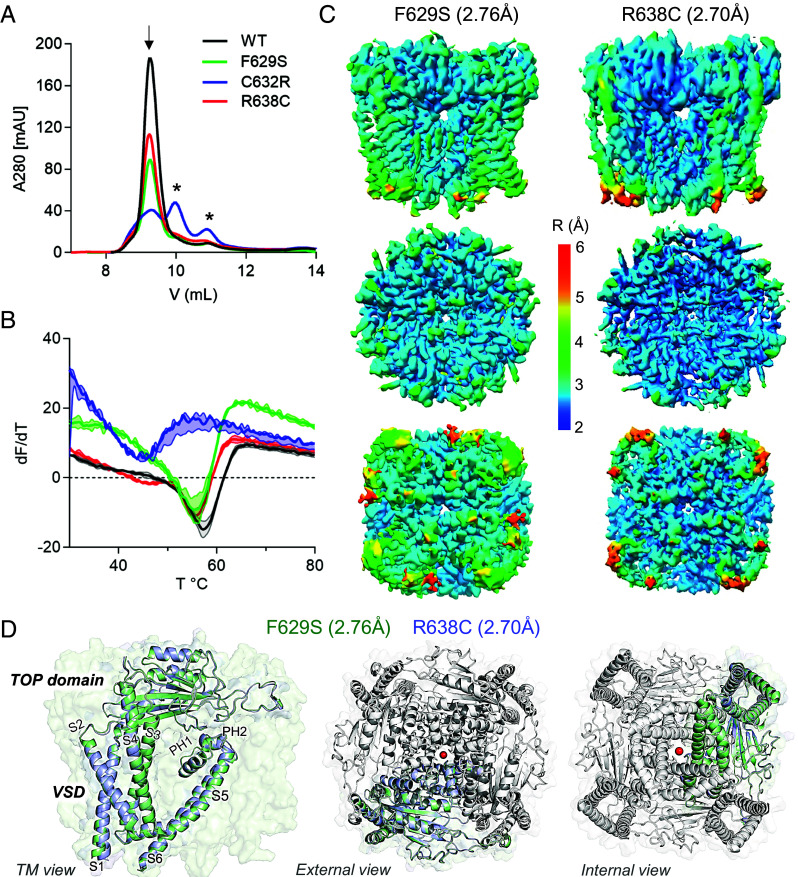
Cryo-EM structural determination of ADPKD-causing PKD2 missense variants and their impact on channel assembly. (*A*) Size exclusion chromatography results of WT and PH1 variant channels purified from expi293F GnTI-/- cells. Peaks indicate the relative abundance of each channel as oligomers (arrow) or protomers (asterisk). (*B*) GlowMelt (Biotium) thermal stability measurement of polycystin protein unfolding. First derivative (slope) of the fluorescence curve (dF/dT) for each channel protein is plotted as a function of temperature (T). Error bars = SD, N = 3 replicates for each channel type. (*C*) Local resolution maps of PKD2 F629S and R638C structures determined by cryo-EM. Note, pore domain resolution is between 2.5 to 3.0 Å and 2.0 to 2.5 Å for the F629S and R638C structures, respectively. (*D*) Overall structure of the variant channels. *Left*, Transmembrane view and structural alignment of the F629S and R638C channels, highlighting the domains within a single-channel subunit. *Right*, External and internal views of the channels demonstrating the domain-swapped assembly of the channel.

**Fig. 2.**
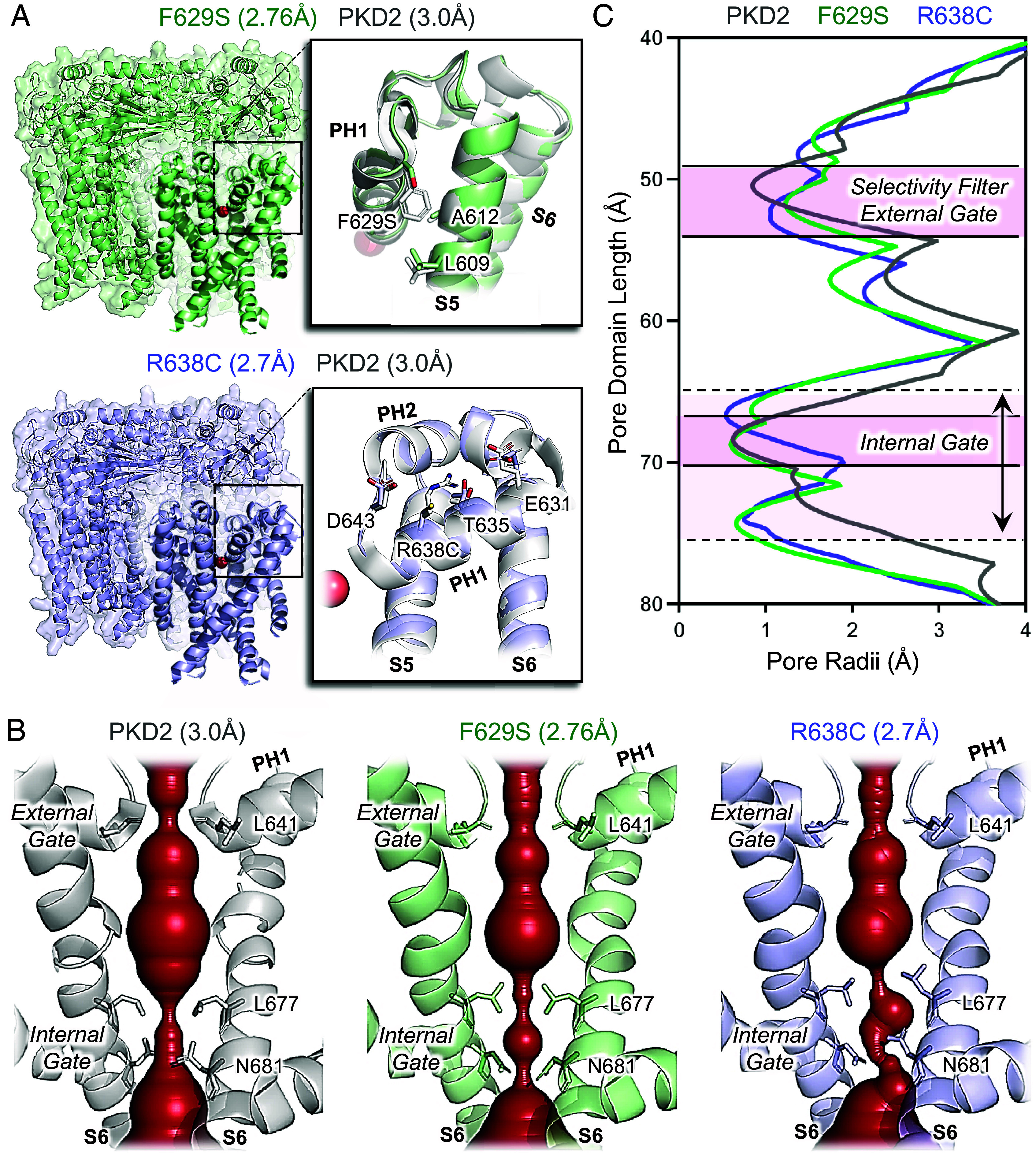
Allosteric structural effect of pore helix variants on the PKD2 ion-conducting pathway. (*A*) Structural alignment of the variant and WT channels [PDB: 5T4D, Shen et al. (18)]. *Inset*, Expanded views of the pore domains showing the location of the ADPKD-associated variants highlighting the missing pore helix chemical interactions. (*B*) HOLE analysis of the PKD2 pore domains highlighting the changes in the internal and external gates (26). (*C*) Analysis of the polycystic pore radii plotted along their length. Note the elongated pore restriction at the internal gates (double pointed arrow) of the F629S and R638C compared to the WT channels.

To biochemically and structurally assess the impact of ADPKD missense variants, we expressed WT and mutated forms of PKD2 in HEK cells and isolated channel protein using DDM (n-dodecyl β-d-maltoside) detergent. The oligomeric assembly of WT, F629S, and R638C protein samples is apparent by their monodispersed elution fractions as evaluated by size-exclusion chromatography (SEC), whereas the C632R variant was prone to disassociation (Fig. 1*A*). This variant-specific destabilizing effect was supported by dye-based protein thermal denaturing experiments performed on the recombinant samples (Fig. 1*B*). Here, the C632R channel was highly unstable at physiological temperatures (37 to 38 °C), given by the derivative change in fluorescence indicating the unfolding of the channel protein, whereas the remaining variants only denatured after heating the samples beyond 50 °C. The heterogeneous and polydisperse characteristics of recombinant C632R samples prevented further structural characterization, and we proceeded to single-particle cryo-EM analysis of the F629S and R638C variants. Between 333 K-665 K particles of the variant channels were extracted, processed, and refined with C4 symmetry to resolve their molecular structures at 2.76 Å (F629S) and 2.70 Å (R638C) GSFSC resolution (Fig. 1*C* and *SI Appendix,* Fig. S2 *A* and *B*) (22). Each channel subunit encodes six transmembrane spanning helices (S1 to S6) which fold into structural domains: voltage sensor (VSD, S1-S4), tetragonal opening for polycystins (TOP), pore (PD, S5 and S6), and cilioplasmic (N and C termini). Like previously resolved WT channel structures, the PKD2 variant subunits formed a domain-swapped tetrameric channel architecture (Fig. 1*D*) (16–19). The VSDs of the F629S and R638C variants structures are captured in the deactivated state with S4 gating charges (K572 and K575) oriented for hydrogen bonding and cation-π interactions that facilitate gating charge transfer (*SI Appendix,* Fig. S3 *A* and *B*) (23). The deactivated VSDs are expected as polycystins are voltage-gated channels that open at positive voltages and the protein samples were prepared without a membrane potential (0 mV) (12, 13, 23, 24). The ion-conducting pathway within the PD of homomeric polycystin channels has two restriction sites which were proposed to function as “gates” (Fig. 2 *A*–*C*) (18, 25). The putative external gate is found within the ion selectivity filter (L641-N643) that is scaffold by two reentrant pore helices (PH1 and PH2), and the internal gate is located at tetrameric S6 junction formed hydrophobic residues (L677). Both gates of F629S and R638C variant channels are captured in nonconducting closed states based on their PDs minimum radii (R_min_ = 0.55 to 0.62 Å) that are too narrow for partially hydrated cations (Na^+^, K^+^, and Ca^2+^) to permeate (Fig. 2 *B* and *C* and *SI Appendix,* Fig. S4 *A*–*C*). As expected, several local PH1 molecular interactions are absent from the F629S and R638C variant structures compared to the previously resolved WT PKD2 channel (Fig. 2*A*) (18). On the lipid-facing side of the PH1 in the F629S channel structure, the large phenyl sidechain density which is normally found buried in a hydrophobic cleft formed by cilia membrane and nonpolar residues (L609 and A612) of the S5 was replaced by an unfavorable polar serine hydroxyl. In the R638C structure, the guanidinium functional group is replaced by a sulfhydryl, disrupting a hydrogen-bond tripartite network formed with carboxylate and hydroxyl sidechains of the PH1 (E631 and T635), and selectivity filter (D643) residues normally found in WT channels. Despite the localized disruptive interactions, the pitch and secondary fold of the PH1 helix are preserved in the F629S and R638C variant structures and only cause a small dilation (ΔR_min_ < 0.24 Å) in the external gate (Fig. 2 *B* and *C*). However, examination of the variant channel’s internal gates reveals key differences in L677 and N681 sidechains rotamers that effectively double the length of its restriction (6 to 8 Å) along the S6 (Fig. 2 *B* and *C*). Here, changes in the orientation N681 carboxamides optimize hydrogen bonding distances (3.4 to 2.8 Å) between each protomer. These interactions stabilize the occluded ion-conducting pathway observed in both structures and create asymmetry observed at the inner gate of the F629S channel (*SI Appendix*, Figs. S3*C* and S4*C*). Based on these biochemical and structural results—ranging from complete channel assembly failure (C632R) to allosteric collapse of the internal gate (F629S and R638C)—we investigated the PH1 variant impacts on channel trafficking and biophysical properties in primary cilia membranes.

### Pore Helix Variants Cause Divergent Effects on Primary Cilia Trafficking and Channel Gating.

**Fig. 3.**
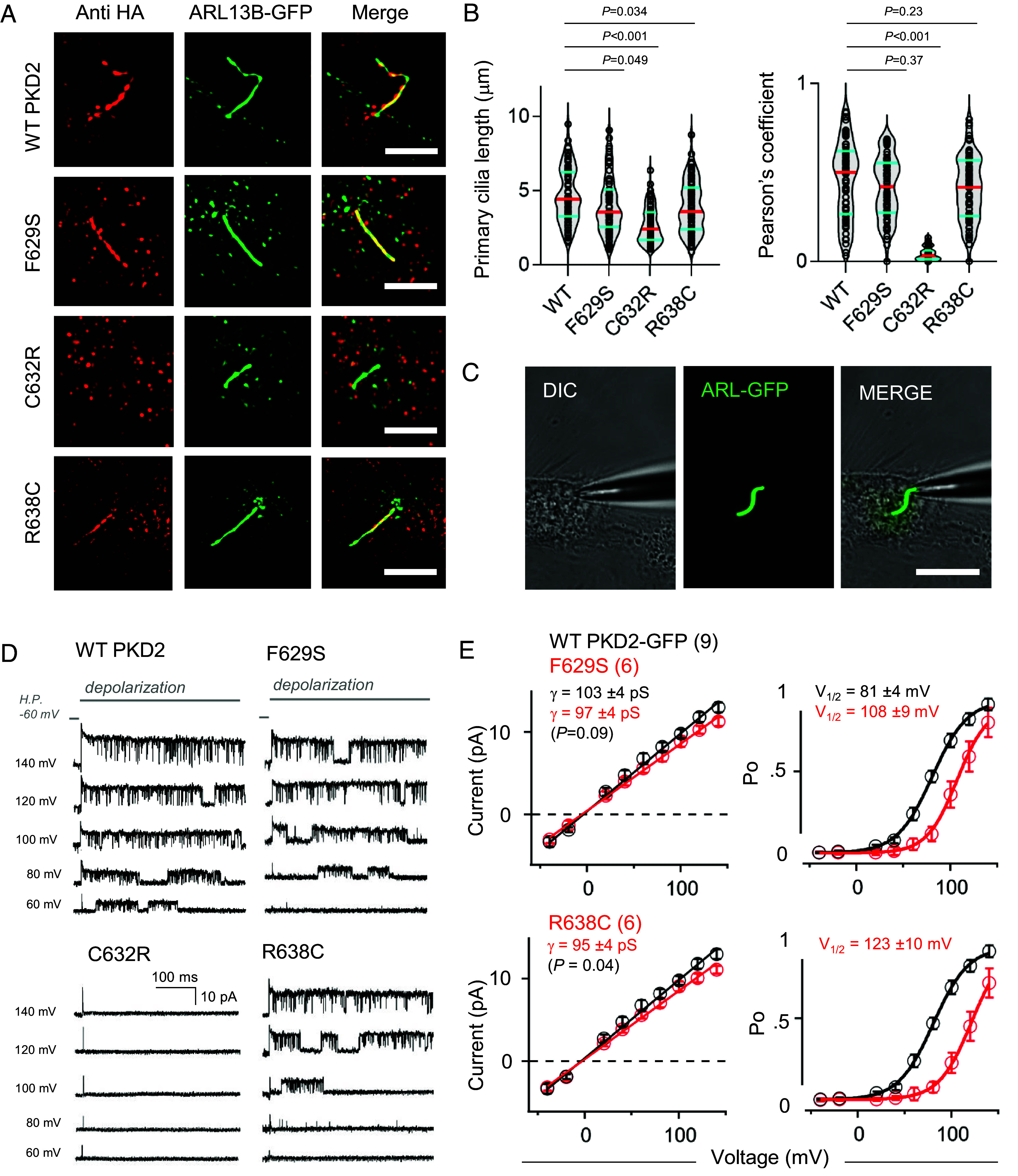
ADPKD-causing PKD2 missense variants cause partial to complete loss of channel function through impaired trafficking defects or impaired gating. (*A*) Superresolution SIM (structured illumination microscopy, Scale bar, 3 μm) images of HEK PKD1^Null^:PKD2^Null^ cells stably expressing ARL13B-GFP (primary cilia reporter) and transiently transfected HA-PKD2 channels immunolabeled with anti-HA antibody (red). (*B*) Primary cilia length and fluorescence colocalization analysis using Pearson’s correlation coefficient. (*C*) Image a voltage clamped HEK PKD1^Null^:PKD2^Null^ cell expressing the PKD2 WT channel with its primary cilium illuminated under fluorescence. (*D*) Example PKD2 WT and variant single-channel records recorded from the cilia and activated by the indicated depolarization steps. (*E*) *Left*, Single-channel current magnitudes comparing WT and variant channels. Unitary conductance was estimated by the slope (γ) of the linear fit. *Right*, Open probability (Po) plotted as a function of voltage and fit to a Boltzmann equation to estimate the slope (Z) and half-activation voltage (V_1/2_). Error bars = SD, and the number of cilia records (N) for each dataset is indicated within the parenthesis. *P*-values indicate results from two-tailed, unpaired Student’s t-tests comparing WT and variant channel datasets.

Using superresolution 3D-structured illumination microscopy (3D-SIM), we compared primary cilia trafficking of the PKD2 pore helix variants. PKD1 and PKD2 null HEK cells (PKD1^Null^:PKD2^Null^) stably expressing a genetically encoded primary cilia reporter (ARL13B-GFP) were transiently transfected with plasmids encoding HA-tagged variant channel prior to fixation, immunolabeling, and colocalization analysis (Fig. 3 *A* and *B*) (20). Expression of the exogenous levels of polycystin protein was equivalent among expressed channels as assessed by western blot analysis from total cell lysates (*SI Appendix*, Fig. S5 *A* and *B*). As expected, misfolded and unassembled C632R channel subunits do not localize to the primary cilia and remain sequestered in the cell. In contrast, the structured and assembled F629S and R638C variants traffic to the ciliary organelle membrane, like WT channels (Fig. 3 *A* and *B*) (22). Overexpression of the PH1 variants significantly impaired primary cilia length in these cells, where the C632R variant had the greatest impact (WT L_cilia_ = 4.7 µm, C632R L_cilia_ = 2.4 µm). To assess the impact of the variants on channel function, we used microelectrodes to form high resistance seals with the primary cilia membrane and performed single-channel voltage clamp recordings (Fig. 3 *C*–*E*) (12). Voltage-dependent channel openings were evident from cilia expressing F629S and R638C channels (Fig. 3*C*). In agreement from the cilia trafficking defects observed by superresolution, no channel opening events were detected from cilia patch clamp recordings from cells expressing the C632R variant (N = 15 cilia) and control cells devoid of polycystin gene expression (N = 18 cilia from HEK PKD1^Null^: PKD2^Null^; *SI Appendix*, Fig. S5*C*). Voltage dependence of opening (V_1/2_) of the F629S and R638C channels were positively shifted (ΔV_1/2_ = 27 to 32 mV) compared to WT, which dramatically increased the free energy of gating (ΔG°) by 133% (+1.2 kcal/mol), and 152% (+1.8 kcal/mol), respectively (Fig. 3*D* and *SI Appendix,* Table S1). The unitary conductance (γ) of both variants was significantly decreased, suggesting a small reduction in the rate of cation permeation through the pore. Taken together, the superresolution imaging and electrophysiology datasets establish divergent loss of channel mechanisms caused by ADPKD variants. While the F629S and R638C variant results in a partial loss of function, producing a significant energy barrier to opening the PKD2 channel, the C632R variant causes a complete loss of cilia localization through its failure to traffic to this organelle.

## Discussion

### Summary of Findings.

Using multidisciplinary methods which include superresolution imaging, single-particle cryo-EM structural determination, and cilia microelectrode electrophysiology— we have defined the molecular mechanism and functional consequences of three PKD2 pathogenic missense variants that cause ADPKD. Even though all three mutations are located within the same PH1 structural motif, they have divergent mechanistic impacts. Through biochemical and 3D-SIM analysis, we determine that C632R causes a catastrophic destabilization of PKD2 oligomeric assembly which precluded its structural determination and functional interrogation. Nonetheless, the impact of C632R for renal cells is a complete loss of function due to the channel’s exclusion from the primary cilia. In contrast, the F629S and R638C variants cause partial loss of function having normal trafficking and assembly, but impaired voltage-dependent gating. The unitary conductance of these variants is also reduced, a feature which adds a second loss of function effect and may also contribute to disease pathology. Conclusions from the primary cilia electrophysiology results are supported by features observed in our F629S and R638C cryo-EM structures. These missense variants break critical PH1 interactions but do not cause catastrophic changes of its secondary structure or the channels’ overall assembly. Rather, the F629S and R638C mutations cause an allosteric collapse in pore-lining S6 at the internal gate—doubling the length of its hydrophobic septum. Our results suggest ADPKD is caused by loss-of-PKD2 function in the primary cilia, which can be driven by impaired channel gating and trafficking defects. Within the context of other channelopathies, pore helix missense variants in many voltage-gated channels are associated with arrhythmogenic Long QT syndrome 1 (Kv11.1, KCNH2 gene); Long QT syndrome 2 (Kv 7.1, KCNQ1 gene); Brugada syndrome (Na_V_1.5, SCN5A gene) several types of epilepsy (Na_V_1.1, SCN1A gene; Na_V_1.3, SCN3A gene); and other conditions (27–31). The mechanistic impact of these mutations are wide ranging—causing gain or loss-of-function in channel gating through altered activation and inactivation kinetics (32–35). A limitation of this study is the unexplored consequence of variants on PKD1-PKD2 heteromeric channels. Future work should define the unknown gating behavior of these heteromers and their cryo-EM structural features when complexes with ADPKD-causing variants. Nonetheless, discussed below are the significance of these findings related to PKD2 channel molecular regulation and the development of polycystin-targeted drugs for the ADPKD patient population.

### Implication for Polycystin Structural Gating Mechanisms and Channel Assembly.

**Fig. 4.**
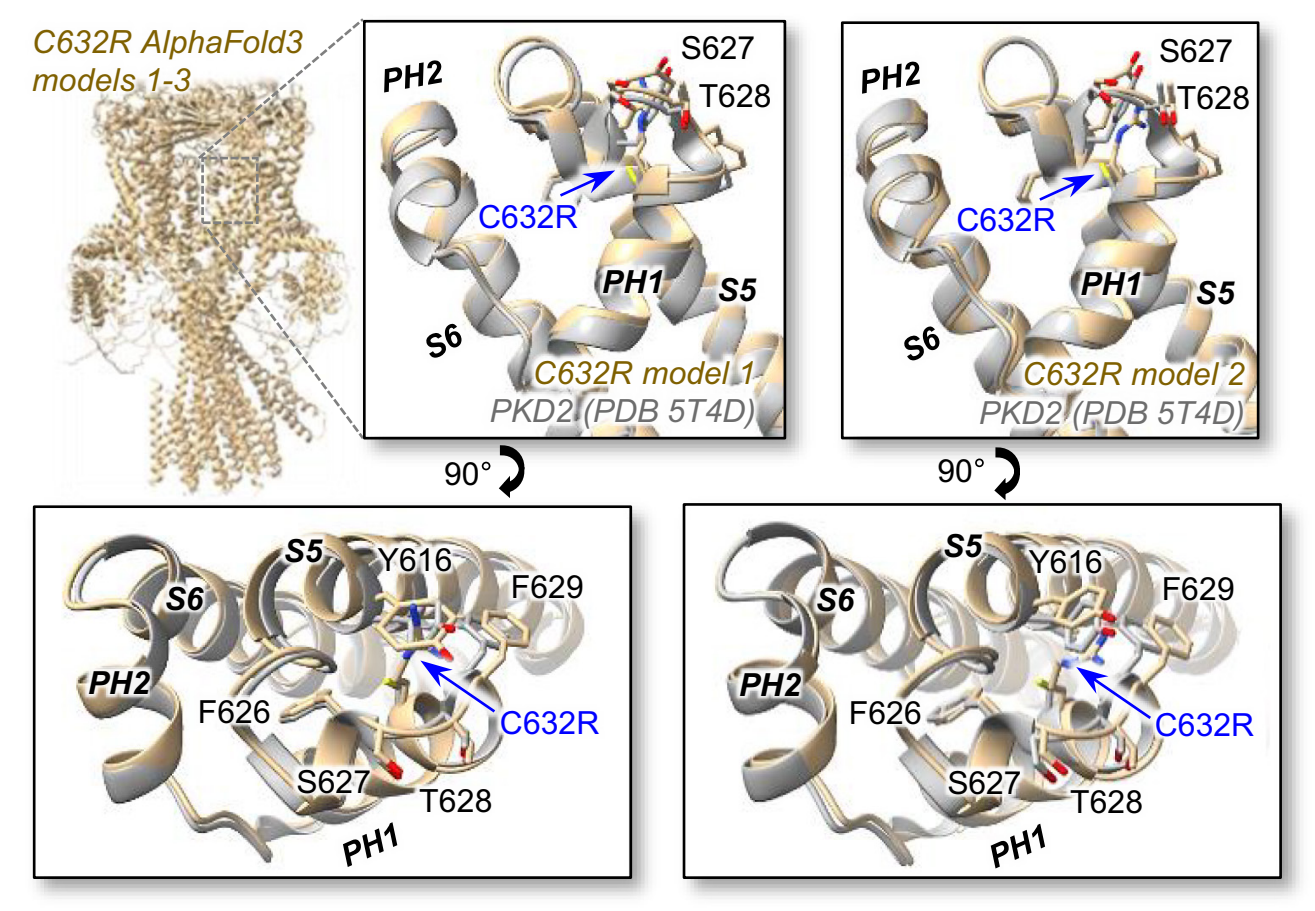
In silico assembled PKD2 C632R channels using AlphaFold3. *Left*, PKD2 C632R channel (residues 180 to 925) modeled using AlphaFold3 as a homotetramer (models 1 to 3). *Right* and *Lower insets*, Structural alignments of the WT PKD2 cryo-EM structure (PDB 5T4D) and the AlphaFold3 models with views of the external pore helices and S5 interface highlighting the steric clashes between C632R and Y616 side chains of models 1 and 2 (18, 41).

The molecular mechanisms controlling opening of the polycystin pore domain are poorly understood. As identified in the original cryo-EM structural determination of PKD2, the pore domain’s ion‐conducting pathway is constricted at internal and external sites, which are proposed to serve as gates (18). While it is widely accepted that the external site is the ion selectivity filter, its putative function as a gate is controversial (25, 36). The role of pore helices as structural regulators of channel opening is further supported by results from an unbiased mutagenic screen of the related PKD2L1 pore domain (37). Although the pore helices scaffold the selectivity filter/external gate, but we did not find significant changes at this site among the pore helix variant structures. While we reported a small dilation (ΔRmin < 0.24 Å) at this site, our interpretation is limited by the resolution of the cryo-EM datasets. PH1 variants caused a reduction of unitary conductance, a functional observation consistent with selectivity filter or pore-lining residue structural defects which may limit the flow of cations through the conducting pathway (36, 38–40). In contrast to the external gate, the variants caused significant changes (lengthening, asymmetry) to the internal gate formed by the crossing of S6 helices. These variant-induced inner gate structural defects were functionally validated by energetically expensive shifts in voltage-dependent gating when recorded from the cilia membrane. Taken together, these results are most consistent with a “one‐gate” mechanism—where long‐range coupling energetics between the pore helices are transferred to the inner gate within the pore. *How can variants within the PH1 alter the inner gate’s function, given the sites are separated by long molecular distances* (20 Å)? The precise allosteric molecular mechanism is not fully understood. However, we propose the upper and lower gates are likely coupled via an interaction network that involves residues at the selectivity filter (F641) and pore helix 1 (R638) of one subunit and pore helix 2 (F646) and the pore-lining S6 helix (F661 and V665) of a neighboring subunit (*SI Appendix*, Fig. S3*B*). The ADPKD R638C variant removes a cation-π interaction between R638 and F646 and reduces the coupling between the pore helices of adjacent subunits. Whereas the polarity of the F629S serine side chain unfavorably dips into a hydrophobic pocket formed by the membrane and nonpolar S6 residues of the same subunit. We hypothesize that this mutation alters the conformational coupling between the VSD and PD and stabilizes the nonconductive state of the pore observed in our F629S cryo-EM structure. Importantly, both variants impact this gating mechanism through discrete chemical interactions, without altering the structure of the local pore helix or the overall quaternary channel assembly. In contrast, the C632R variant destabilized oligomeric assembly which barred its structural determination. To explore the molecular cause, we generated hypothetical models of the C632R channel using AlphaFold3 (DeepMind) (22, 41). The protein folding problem induced by the missense variant clearly challenged the AI system—as the C632R channel required significantly more time (72 more hours, under 1 GPU processing power) to render semicoherent structures compared to the native channel. The resulting models identify steric clashes (not chemically possible) created by the substituted arginine side chain at site C632 with residue Y616 emanating from the S5 (Fig. 4). Evidently, the in silico modeling results mirror our in vitro biochemical findings, supporting the hypothesis that the C632R variant uniquely causes devastating impairment of channel formation. Taken together, our study of the PH1 variants has elucidated the unexpected biophysics governing channel gating, conductivity, and channel assembly—and provides a mechanistic explanation for their loss of function that drives ADPKD progression.

### Context of Findings Related to Previously Published Work.

PKD2 forms homotetrameric voltage-dependent and calcium modulated ion channels in primary cilia membranes (13, 42–44). For reasons which are not fully understood, PKD2 expression does not contribute to plasma membrane currents—both under endogenous expression in primary renal collecting duct cells and when overexpressed in mammalian cells (11, 12, 18). Because PKD2 contributions to plasma membrane currents in *Xenopus* oocytes is controversial, several groups have included the F604P gain-of-function mutation (no disease relevance) into channels when evaluating the effects of disease-causing variants (45–48). This drastic mutation “breaks the gate” of PKD2 by uncoiling the S6 π- to α-helix transition which displaces the lower gate (L677) out of the ion-conducting pathway, as resolved in cryo-EM datasets (45, 49). As a functional consequence, channels expressing the F604P mutation produce robust constitutive current with no voltage-dependent gating kinetics (45, 49). Thus, a major caveat of this approach is the loss of native structural and biophysical properties typically observed in PKD2 channels recorded from primary cilia membranes. Furthermore, loss-of-function effects resulting from disease-causing variants could be masked by the gating defects conferred by the F604P gain-of-function mutation. Nonetheless, as it directly relates to our study, double mutant channels expressing both the F604P with pore helix variants (F629S, C632R, and R638C) result in net loss of current without impacting the level of plasma membrane expression in oocytes (50). These results partly conflict with the findings reported in this manuscript. While we report F629S and R638C variants result in loss of channel function through impaired gating, our biochemical analysis of the C632R variant clearly demonstrates that this mutation abolishes channel assembly, which naturally results in loss of channel trafficking to the primary cilia. Since our methodologies were conducted using kidney cells expressing PKD2 with single-pore helix mutations, we suspect the differences in results are related to the former study’s use of unciliated amphibian expression systems and the pore-rearrangement induced by the inclusion of the additional F604P mutation. Since ADPKD is considered a renal ciliopathy–channelopathy, and because ciliary exclusion of PKD2 promotes kidney cystogenesis, our results were obtained from a purposefully designed experiment that assesses channel trafficking and functional defects in their near-physiological context (51–53). In this study, cilia length is negatively impacted by overexpressing PKD2 variants compared to WT channels in immortalized PKD1^null^:PKD2^null^ HEK cells. Previously, we did not see differences in cilia length from cells expressing partial loss-of-function in the PKD2 TOP domain variants (20). However, those experiments were carried out in PKD2^null^ HEK cells. Thus, the difference in results might be related to complete removal of background renal polycystin gene expression (PKD1 and PKD2). We found that primary cilia shortening correlated with severity of the variants impact on channel trafficking—with cells expressing the C632R having the shortest cilia. These results suggest functional polycystin expression is correlated with primary cilia stabilization and/or formation. While this observation supports the channelopathy-ciliopathy categorization of ADPKD, further studies are needed to define the role of polycystins and their downstream mediators responsible for cilia morphology defects. Although we found the overall protein expression of PKD2 was not impacted by pore helix mutations using western blot analysis, we did not evaluate their impact on protein content in the primary cilia or other cell membranes. Future work using newly developed methods such as surface scanning electron microscopy combined with immunolabeling (immuno-SEM) may provide new ways to assess variant-induced changes in cilia PKD2 protein levels (54).

### Impact on the Rationale for Polycystin-targeted ADPKD Drug Development.

There is currently no drug cure for ADPKD. However, development of pharmaceuticals which activate variant polycystins or reinstating their channel function may present a viable therapeutic strategy. This view is supported by in vivo studies in which derepression or rescue of polycystin gene expression ameliorates cyst growth in mouse models of ADPKD (55, 56). These results suggest pharmacological activation of PKD1 or PKD2 is sufficient to attenuate disease progression. We have determined the PKD2 variants F629S and R638C traffic to the primary cilia but have defective gating properties and reduced ion conductivity. Based on our findings, activating these functional and cilia-localized polycystins using prototypic channel modulators may be a viable drug strategy. Alternatively, reinstating PKD2 C632R trafficking through the design of molecular chaperones (“correctors”) that stabilize the channel protein may also prove beneficial. Proof of concept of this approach is demonstrated by VX-407, a prototypic drug under clinical trials, which corrects defective PKD1 folding within a subset of variants. However, the efficacy of this drug against the ADPKD population with PKD2 variants is unreported. Further elucidation of mechanistic differences among disease-causing variants and the polycystin pharmacophore is needed to evaluate prototypic pharmaceutics for the genetically diverse ADPKD patient cohort. Alternatively, developing patient genotype-specific (i.e., variant-targeted) therapeutics may inform a personalized medicine approach as a drug development strategy.

## Methods

### Expression, Purification, and Thermal Denaturing of PKD2 Channels.

PKD2 WT and point mutant variants (F629S, C632R, R638C) were expressed by transient transfection in Expi293F GnTl^−/−^ cells using an N-terminal Strep-tagged MBP-TEV-PKD2 construct (hPKD2: 52-793) under control of a CMV + β-globin intron promoter. Expi293F GnTl^−/−^ cells were grown at 37 °C to 3 × 10^6^ cells/mL density and transfected with PEIMax (DNA:PEIMax 1:5). 20 to 22 h posttransfection, sodium butyrate was added to boost protein expression (10 mM). Cells were harvested 72 h posttransfection, washed with PBS, and stored at −80 °C until further use. Cell pellets were resuspended in Buffer A (25 mM HEPES pH = 7.4, 150 mM NaCl, 1 mM TCEP, 1 mM CaCl_2_, and 10% glycerol) supplemented with complete protease inhibitors. The cells were homogenized by sonication at 30% power and supplemented with n-dodecyl-β-D-maltoside (DDM) in Buffer A to a final concentration of 2% w/v. Membranes were solubilized by gentle nutation at 4 °C for 2 h, and cell debris was removed by centrifugation at 20,000 g for 30 min at 4 °C. The cleared lysate was applied to 0.5 ml of equilibrated Strep-Tactin Superflow affinity resin by gravity flow. The column was washed with 30 CV of Buffer A + 0.05% DDM/0.005% CHS and eluted in 5 CV of Buffer A supplemented with 5 mM D-desthiobiotin. Protein concentration was estimated by A280 followed by supplementation with amphipol A8-35 (1:3 w/w) and incubation for 1 h at 4 °C. Detergent was removed with two batches of SM2 Biobeads (10 mg/0.1 mL) by gentle nutation at 4 °C, first for 1 h and then overnight. The MBP solubility tag was cleaved simultaneously with the last batch of Biobeads by the addition of TEV Protease. PKD2 52-793 reconstituted in A8-35 was polished by size exclusion chromatography in a Superdex 200 10/300 GL column using Buffer B (25 mM pH = 7.4, 150 mM NaCl, 1 mM TCEP, and 1 mM CaCl_2_). Peak fractions eluting at ~9.5 mL postinjection were pooled, concentrated with 100 kDa cutoff centrifugation filters, and used for CryoEM sample preparation.

### Protein Thermal Denaturation.

Thermal shift experiments were performed in a Thermo Fisher QuantStudio 7 qPCR instrument at 2 °C/min temperature ramp from 20 °C to 95 °C using MicroAmp Fast 96-well PCR plates. 10 µg of SEC purified PKD2 variants in A8-35 were mixed to a final concentration of 1X GloMelt dye (Biotium 33021-T) and 50 nM ROX dye internal standard in 20 µL of buffer (25 mM HEPES pH =7.4, 150 mM NaCl, and 1 mM CaCl_2_). Thermal denaturing experiments were performed in technical triplicates and in the absence of reducing agents. The denaturation temperature for a specific variant is given by the dF/dT peak minimum.

### Cryo-EM Sample Preparation and Data Collection.

Samples were plunge frozen in a Leica EM GP2 at 5 °C and >95% humidity and −183 °C liquid ethane temperature. PKD2 (53-792) variants reconstituted in amphipol A8-35 (0.5 to 1.0 mg/mL) were applied to a glow discharged (air, 15 mA, 20 s) Quantifoil Holey Carbon 2/1 copper 300 mesh grids. 3.5 µL of sample was applied to the carbon side, and the grid was immediately blotted for 2.5 to 3.5 s at 42 mm/2 mm blot distance. Preliminary grid screening and data collection were conducted at the Pacific Northwest Cryo-EM Center (PNCC) using SerialEM software. Datasets were collected on a Titan Krios G3i equipped with a K3 detector and energy filter (10 eV) slit. Movie stacks composed of 50 subframes and 60 e^−^/Å total dose were collected at 130,000× magnification on superresolution mode (0.3235 Å pixel size). The target defocus acquisition range was set between −0.6 and −2.4 µm.

### Cryo-EM Data Processing.

Data processing was performed in CryoSPARC, including the initial preprocessing motion correction, dose weighing and CTF estimation, and micrograph curation steps.

PKD2 R638C Variant: 1,6586 movies were collected. 750 particles from 40 micrographs were picked manually to create initial templates. These templates were used to auto-pick particles from 1,000 micrographs and create new templates. The second generation of templates was used to auto-pick the full 1,4292 curated set of micrographs. 2,084,635 particles were extracted with an 864-pixel box size, down-sampled to 432 pixels, and subjected to two rounds of 2D classification (50 classes). The top 12 classes (665,417 particles) were selected and used to create an ab-initio volume. Homogeneous refinement imposing C4 symmetry yielded 2.7 Å GSFSC resolution reconstruction.

PKD2 F629S Variant: 9,145 movie stacks were collected. The 7,331 curated micrograph stack was used for blob picking polycystin particles from denoised micrographs (130 to 170 Å diameter range). 1,046,126 particle picks were extracted with an 800-pixel box size, down-sampled to 400 pixels and subjected to two rounds of 2D classification (60 classes). The top 17 class averages (333,223 particles) were selected and used to create 3 ab-initio reconstructions followed by heterogenous refinement to parse particles corresponding to the PKD2 tetrameric assembly. Homogenous refinement imposing C4 symmetry yielded a 2.7 Å GSFSC resolution reconstruction. B-factor sharpening 107.9).

### Model Building and Structure Validation.

AlphaFold2 models (PKD2 residues 180-925) containing the variant of interest were pruned in PHENIX (57) to remove low-confidence residues. The resulting PKD2 variant models, composed of residues E214-Q694, were manually fitted into the Cryo-EM map using ChimeraX (58). Model refinement and validation was performed using ISOLDE (59) which led to the additional pruning of the TOP domain loop (Residues M295-A303), because they cannot be adequately modeled. The structural models from ISOLDE were subjected to a final round of Real Space Refinement in PHENIX before reevaluating structure validation metrics with MolProbity and visualizing individual residue outliers with ISOLDE.

### Generation of HEK Polycystin Null Cell Lines Stably Expressing the ARL13B-GFP Cilia Label.

Using our previously generated CRISPR/Cas9 gene-edited HEK PKD2^null^ cell line, we introduced nonsense mutations to both PKD1 alleles, to create a PKD1^null^:PKD2^null^ cell line. HEK 293 PKD2^null^ cells were electrotransfected with PKD1 sgRNAs (caccGCATAGGTGTGGTTGGCAGC and aaacGCTGCCAACCACACCTATGC) with the All-in-one Cas9 plasmid (Addgene). Cells generated from single-cell clones were selected after 4 wk of expansion under puromycin selection in a 96-well plate. A HEK PKD1^Null^:PKD2^Null^ clone was verified by extracting the genomic DNA and sequencing for the introduced STOP codons within PKD1 and PKD2 genes using the following primers: PKD1 fwd (CTGATGGCTTAGGCCCCTACTG); PKD1 rev (CCTGGGTCTCGGTAGATGAACG); PKD2 fwd (AGCCTCAGGGCACAGAACAG); and PKD2 rev (CCACACTGCCCTTCATTGGC). Plasmids for mammalian expression of ADP Ribosylation Factor Like GTPase 13B C-terminally tagged with GFP (ARL13B-GFP) were custom synthesized (Vector Builder) and subcloned into third-generation lentiviral packaging plasmids used for stable expression contained: pMDLg/pRRE (Addgene), reverse transcriptase pRSV-Rev (Addgene), and envelope-expressing plasmid pMD2.G (Addgene). LentiX-293 T cells (Takara) were transfected with polyethyleneimine (Polysciences) at a 4:1:1:1 ratio of the transgene and viral packaging constructs. Supernatants were collected 48 and 72 h posttransfection and filtered through a 0.45 µm syringe filter. Lentiviral supernatant was concentrated 100 times using 1 volume of PEG-it (System Biosciences) virus precipitation solution and 4 volume of lentivirus-containing supernatant. The PEG-it and supernatant mixture were kept at 4 degrees celsius for 24 h and centrifuged at 1,500 rpm for 30 min. The pellet containing lentivirus was resuspended with 1/100th volume of PBS of the original supernatant volume. Cells were infected with the lentivirus supernatant; ARL13B-GFP expression was selected using culture media containing puromycin (2 μg/ml) for 30 to 90 d. Cells were then fluorescence-activated cell sorted (BD FacsMelody) at 5,000 to 10,000 counts per minute to enrich for the transgene expression. Stable cell lines were cultured in Dulbecco’s modified essential medium (DMEM) supplemented with 10% fetal bovine serum (FBS) and 100 units/ml penicillin, 100 units/ml streptomycin, and 1 μg/ml puromycin selection antibiotic. Expression plasmids encoding N-terminal HA-tagged (hemagglutinin, YPYDVPDYA) human PKD2 were created using the Gibson assembly method. Missense variants were generated using standard, site-directed mutagenesis. Cells were transfected (Lipofectamine 2,000, Invitrogen) 24 to 48 h prior to electrophysiology and superresolution experiments.

### Structure Illumination Microscopy.

PKD1^Null^:PKD2^Null^ cell lines stably expressing the ARL13B-GFP cilia label and transiently transfected with HA-PKD2 channels were fixed with 4% paraformaldehyde (PFA), permeabilized with 0.2% Triton X-100, and blocked by 10% bovine serum albumin in PBS. Cells and tissue were mounted on glass slides and treated with Fluoroshield from Sigma-Aldrich (St. Louis, MO). Primary cilia localization of the variant channels within were visualized by [1/1,000] treated with anti-HA (rabbit) antibodies conjugated with DyLight™ 680 (600-444-384, Rockland). Superresolution images were obtained using an inverted N-SIM Nikon microscope configured for in the Deep 3D-SIM imaging with a 60x silicon oil immersion, 1.3 N.A. objective. Superresolution images using the SIM method were captured under 100× magnification with piezo stepping. Confocal images were further processed with FIJI ImageJ (NIH) and Imaris 9.3 (Oxford Instruments).

### Western Blot Protein Expression.

HEK293 PKD1^Null^:PKD2^Null^ cell line was transfected with N-terminally HA-tagged variant channels using Lipofectamine 2,000. Cells were harvested after 24 h and washed with cold 1X PBS. The cells were resuspended in lysis buffer (25 mM HEPES pH 7.4, 150 mM NaCl, 1 mM TCEP, 1 mM EGTA, 5% glycerol, and complete protease inhibitors) and supplemented with Triton X-100 to a final concentration of 1% (v/v). Cells were gently nutated at 4 °C for 30 min. Cell debris was separated at 15,000 g for 15 min. Total protein concentration in the cleared lysate was estimated by the Bradford assay. 15 µg of total protein was loaded per well into 4 to 20% Tris-Glycine gels. Transfer was performed using a BIORAD Trans-Turbo Blot instrument using 0.2 µm nitrocellulose membranes. Primary antibodies for anti-HA (Thermo # 26183) at 1:5,000 and anti-β-actin at 1:10,000 (Thermo # MA5-15739) were incubated overnight at 4 °C, followed by a fluorescent secondary anti-mouse antibody at 1:5,000 (Thermo # 35502) for 1 h at room temperature. Immunodetection was performed on an IBright 1500 imaging system (Invitrogen).

### Electrophysiology Recordings of Polycystins from Primary Cilia Membranes.

All research chemicals used for our electrophysiology experiments were purchased and supplied by Millipore-Sigma. Voltage clamp experiments capturing transiently transfected PKD2 whole cell currents were made from PKD1^Null^:PKD2^Null^ cell lines stably expressing the ARL13B-GFP cilia label. Cells were seeded onto glass coverslips and placed in a perfusion chamber for voltage clamp recordings in the on-cilia configuration (15). Ciliary PKD2 single-channel events were recorded using borosilicate glass electrodes polished to resistances of 14 to 22 MΩ. The pipette solution contained (in mM): 90 NaMES, 10 NaCl, 10 HEPES, and 10 Na4-BAPTA [Glycine, N, N’-(1,2-ethanediylbis(oxy-2, 1-phenylene))bis(N-(carboxymethyl))-, tetrasodium]; pH was adjusted to 7.3 using NaOH. The bath solution contained 120 KCl, 20 NaCl, 10 HEPES, and 1.8 CaCl_2_, pH 7.4, with NaOH to neutralize the resting membrane potential. All solutions were osmotically balanced to 295 (±6) mOsm with mannitol. Data were collected using an Axopatch 200B patch clamp amplifier, Digidata 1440A, and pClamp 10 software. Currents were digitized at 25 kHz and low pass filtered at 10 kHz. Cilia membranes were held at −60 mV and activated by 1.5 s voltage steps mV from −40 mV to 160 mV at successive +20 mV steps. Data were analyzed by Igor Pro 7.00 (Wavemetrics, Lake Oswego, OR). Open probability–voltage relationships were fit using the previously described Boltzmann equation. Cilia unitary conductance (γ) was estimated by fitting the single-channel current to a linear equation f(x) = γ (Vm)+b. The amount of free energy (G°) required to open WT and variant channels was calculated using this equation: G° = z(F)V_1/2_, where F is Faraday’s number, and z is the estimated charge-based slope derived from the constant.

### AlphaFold Modeling of the PKD2 C632R Variant.

AlphaFold modeling was performed at the Northwestern Structural Biology Facility (SBF) Computing Cluster (41). The C632R mutant sequence was trimmed down to residues 180 to 925 to reduce computational burden by removing unstructured residues in the PKD2 termini.

### Statistical Analysis.

The statistical methods used to assess significance are detailed in the corresponding figure legends and presented as *P* values. Data variance and distribution was assessed using GraphPad or Origin software, employing one-way ANOVA, Student’s two-tailed paired t-tests for equal sample sizes, or unpaired *t* test with Welch’s correction for unequal sample sizes.

## Supplementary Material

Appendix 01 (PDF)

## Data Availability

Raw data, structural coordinates, PDBs, Validation reports data have been deposited in NU library repository ARCH; RCSB PDB (https://doi.org/10.21985/n2-exhe-vs08) (22); The atomic coordinates for the PKD2 F629S and R638C variant channels have been deposited in the protein databank under accension codes PDB 9DWQ (EMDB entry ID EMD-47260) (60) and 9DLI (EMDB entry EMD-46979) (61), respectively.
