# Supplementary material for: Pathogenic variants in the polycystin pore helix cause distinct forms of channel dysfunction
Source: Proc Natl Acad Sci U S A. 2025 Jun 12;122(24):e2421362122. doi: 10.1073/pnas.2421362122 (PMC12184499; doi:10.1073/pnas.2421362122)
Supplement: Supplementary file 1 — Appendix 01 (PDF) [file pnas.2421362122.sapp.pdf]

PNAS

Supporting Information for
Pathogenic variants in the polycystin pore helix cause distinct forms of channel dysfunction.

Orhi Esarte Palomero, Eduardo Guadarrama and Paul G. DeCaen

Corresponding author, Paul G DeCaen
Email: paul.decaen@northwestern.edu

This PDF file includes:

Figures S1 to S5
Tables S1

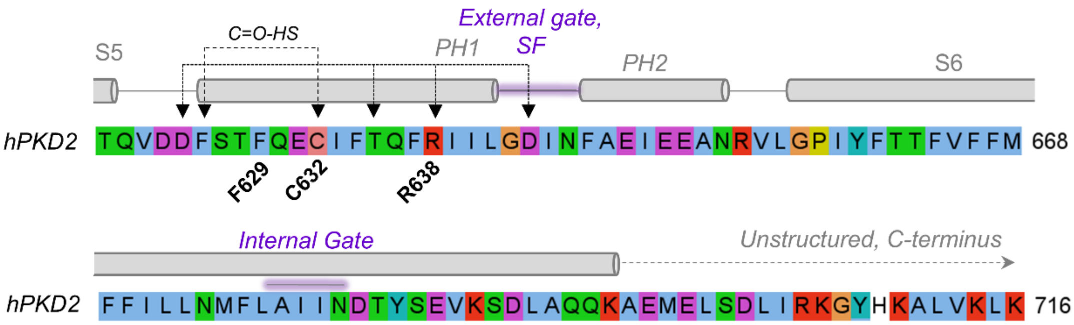

***SI Appendix*, Figure S1. Location of ADPKD-causeing PKD2 missense variants in the pore helix 1.** An amino acid sequence rendered in JalView applying the default color scheme: hydrophobic (blue); polar (green); glutamine, glutamate, aspartate (magenta). Special amino acids are designated with their own color: glycine (orange); proline (yellow) and tyrosine or histidine (cyan). The barrels indicate alpha helices found in the PKD2 structure (PDB: 5T4D) and conserved salt-bridge/hydrogen bonds with the external pore domain are indicated by connecting arrows. Location of PKD2 variants associated with ADPKD are indicated in bold print.

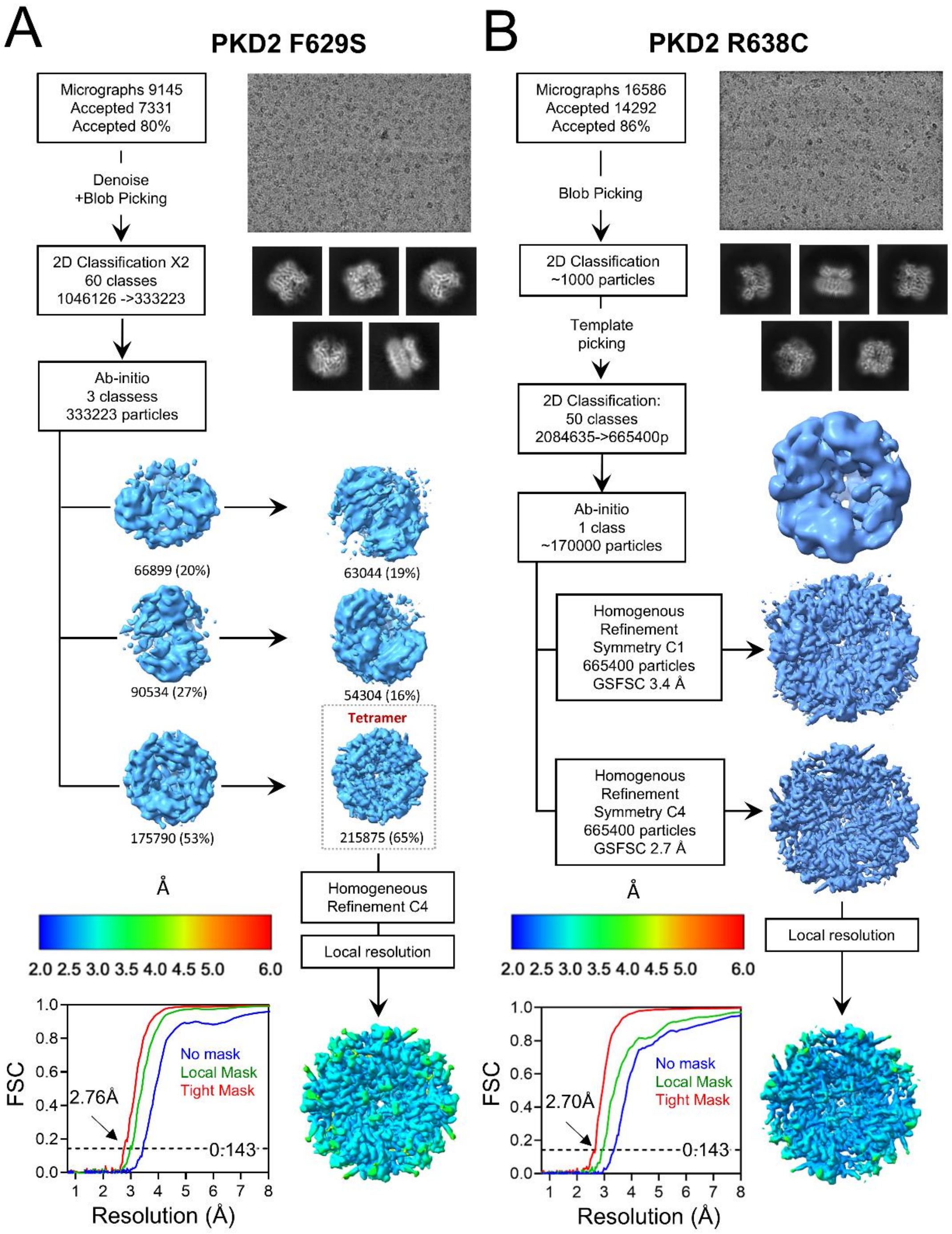

***SI Appendix*, Figure S2. Cryo-EM data processing workflows used to determine the PKD2 F629S and R638C variant structures.** Flow charts outlining the cryo-EM data collection and analysis workflow. Representative micrographs, 2D class averages, and 3D volumes. After homogenous refinement, local resolution maps were generated to build and refine models (See methods section).

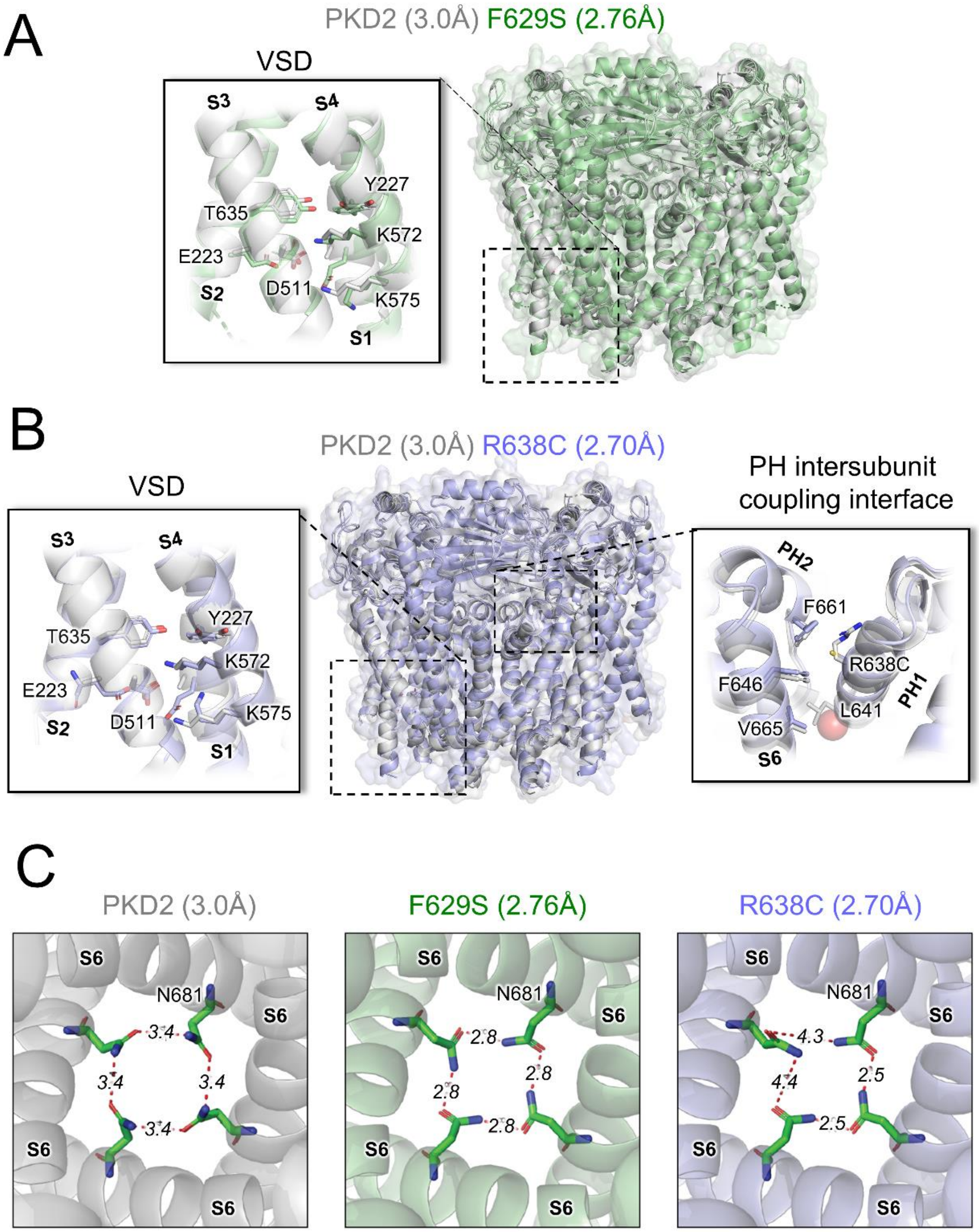

SI Appendix, Figure S3. Key structural features of the PKD2 F629S and R638C varient channels. **A, B**) Transmembrane views of the voltage sensor domains with the gating charges and interacting hydrogen bond partners highlighted. Note gating charges in the varinat and Wt channel structures are in the deactivated state, below the gating charge transfer center. Right, the pore helix intersubunit coupling interface which is likely affected during opening of the R638C varianst channels. **C**) Intercellular view of the homotypic N681 hydrogen bond arrangement for the WT and varainst structures.

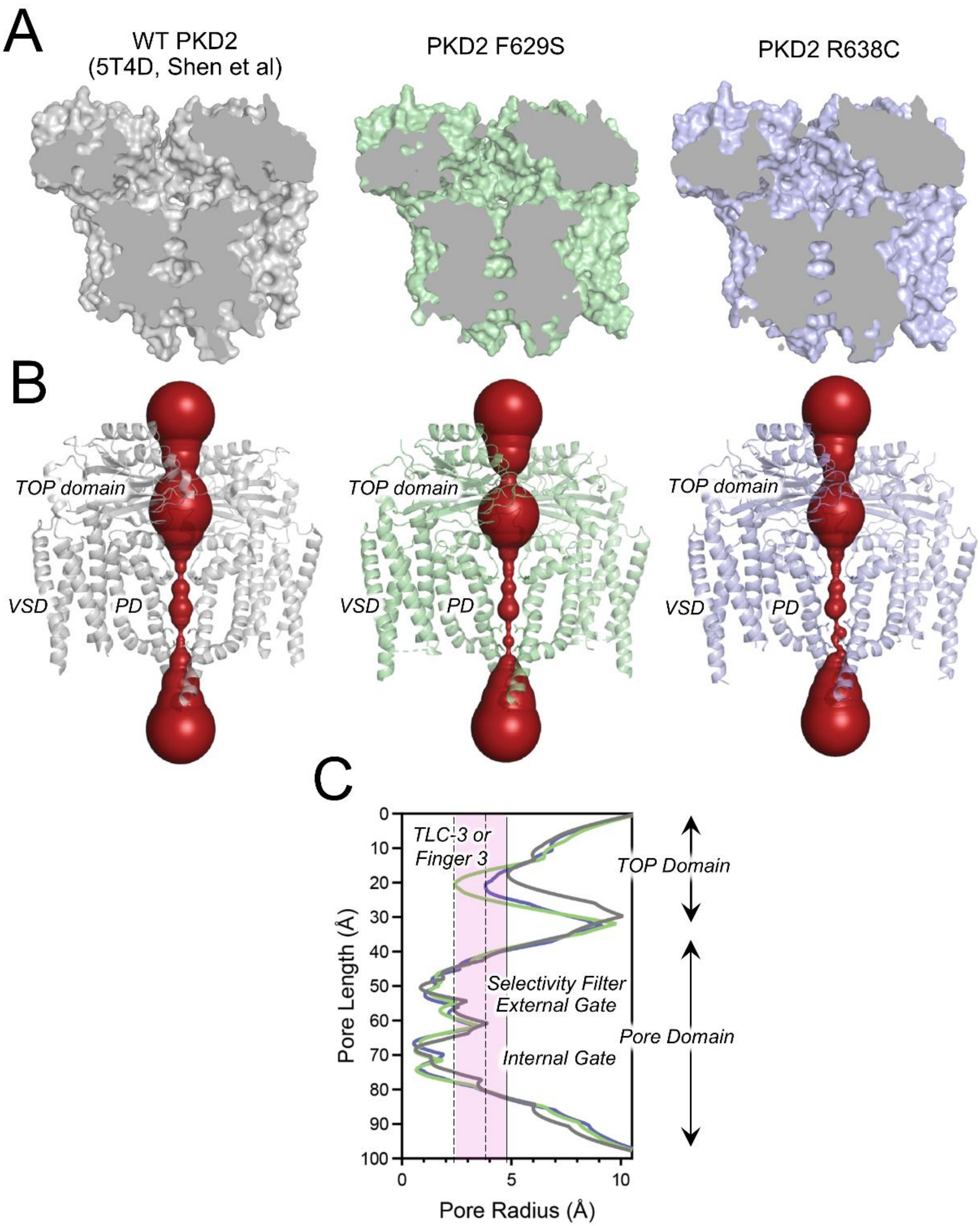

SI Appendix, Figure S4. Analysis of the ion conducting pathway in the cryo-EM varaint structures. **A**) Comparision of the ion condcuting pathway from bisected WT and variant polycystin channel structures. **B, C**) Expanding view (TOP domain to pore domain) of the HOLE analysis results and the pore radius-length plots comparing the WT and variant channels illustrating the changes in the TOP domains.

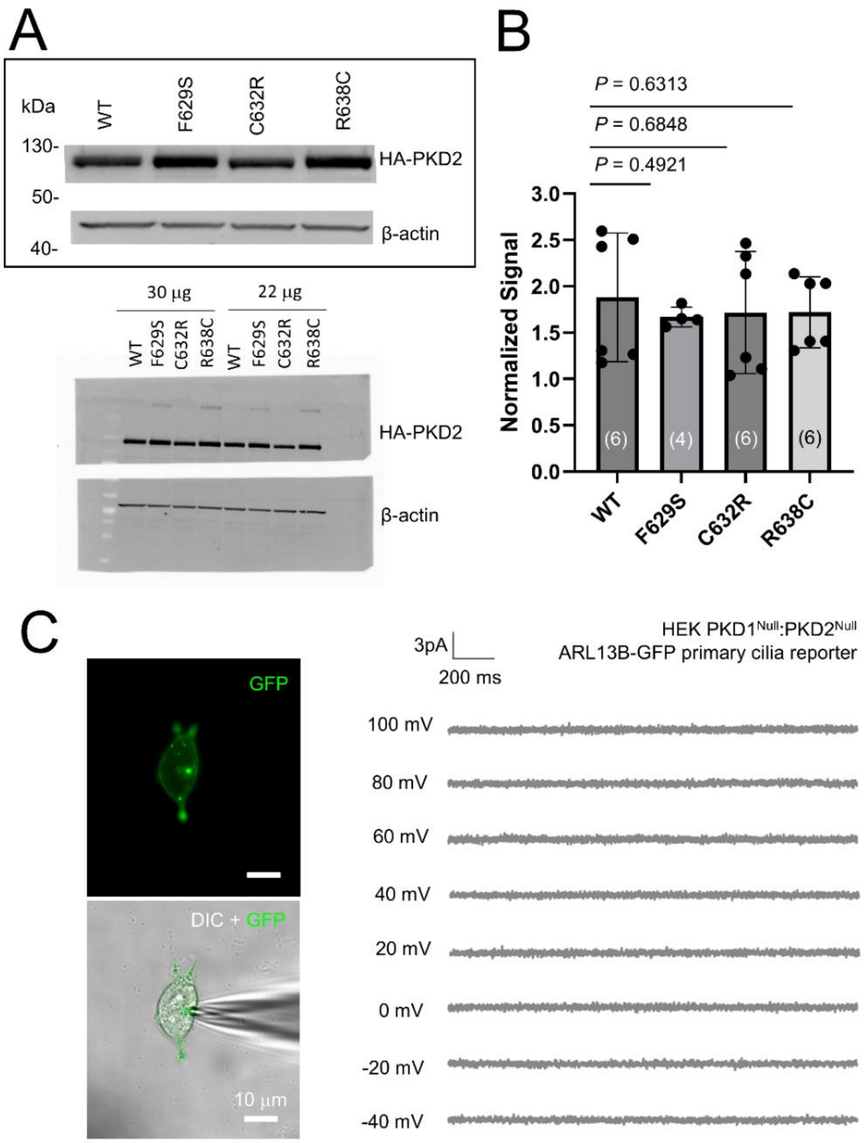

***SI Appendix,* Figure S5. Western blot analysis confirming polycystin protein expression and negative control cilia electrophysiology results.** **A**) Exemplar, western blot taken from HEK PKD1^{Null}:PKD2^{Null} cells expressing exogenous HA-PKD2 channels 32 hours after transfection. The two blots were developed after preparing sample with in indicated amount of total protein. Inset, expanded view of the anti HA signal compared to beta-actin from the 30 μg protein blot. **B**) Western blot analysis of the anti-HA (PKD2) band intensity normalized to the B-actin control protein expression. Mean is indicated at top of the bar graph with and error bar equaling S.D. N = biological replicates is indicated for each group in the parathesis. P values result from an unpaired t-test test with Welch's correction. **C**) Example image and current profiles from voltage clamped primary cilia membrane from PKD1^{Null}:PKD2^{Null} stably expressing the genetically encoded primary cilia reporter (ARL13B-GFP). Note, no single channel currents were detected (N= 18 cilia) in the absence of polycytsin gene expression.

***SI Appendix*, Table S1. Gating properties of PKD2 ADPKD PH1 variants.** Boltzmann parameters ($V_{1/2}$) and (Z) resulting from fitting the voltage-dependent opening relationship of PKD2 channels reported in Figure 3. G° indicates free energy of channel opening quantified by the Gibbs free energy equation described in the methods section. ΔG° indicates the change in free energy induced by the variant. Error = S.D.

**Channel**	**$V_{1/2}$ (mV)**	**Z (qe)**	**G° (kcal/mol)**	**ΔG° (kcal/mol)**
WT	81 ± 3.8	1.91 ± 0.06	3.54 ± 0.6	---
F629S	108 ± 8.6	1.89 ± 0.07	4.70 ± 0.8	1.15
C632R	*No current*			
R638C	123 ± 10.4	1.90 ± 0.08	5.38 ± 1.1	1.84
